# Machine‐learning models for shoulder rehabilitation exercises classification using a wearable system

**DOI:** 10.1002/ksa.12431

**Published:** 2024-08-18

**Authors:** Martina Sassi, Arianna Carnevale, Matilde Mancuso, Emiliano Schena, Leandro Pecchia, Umile Giuseppe Longo

**Affiliations:** ^1^ Fondazione Policlinico Universitario Campus Bio‐Medico di Roma Rome Italy; ^2^ Department of Engineering, Unit of Intelligent Health Technologies, Sustainable Design Management and Assessment Università Campus Bio‐Medico di Roma Rome Italy; ^3^ Laboratory of Measurement and Biomedical Instrumentation, Department of Engineering Università Campus Bio‐Medico di Roma Rome Italy; ^4^ Research Unit of Orthopaedic and Trauma Surgery, Department of Medicine and Surgery Università Campus Bio‐Medico di Roma Rome Italy

**Keywords:** classification, machine learning, rehabilitation exercises, shoulder, wearable sensors

## Abstract

**Purpose:**

The objective of this study is to train and test machine‐learning (ML) models to automatically classify shoulder rehabilitation exercises.

**Methods:**

The cohort included both healthy and patients with rotator‐cuff (RC) tears. All participants performed six shoulder rehabilitation exercises, following guidelines developed by the American Society of Shoulder and Elbow Therapists. Each exercise was repeated six times, while wearing a wearable system equipped with three magneto‐inertial sensors. Six supervised machine‐learning models (*k*‐Nearest Neighbours, Support Vector Machine, Decision Tree, Random Forest (RF), Logistic Regression and Adaptive Boosting) were trained for the classification. The algorithms' ability to accurately classify exercise activities was evaluated using the nested cross‐validation method, with different combinations of outer and inner folds.

**Results:**

A total of 19 healthy subjects and 17 patients with complete RC tears were enroled in the study. The highest classification performances were achieved by the RF classifier, with an accuracy of 89.91% and an F1‐score of 89.89%.

**Conclusion:**

The results of this study highlight the feasibility and effectiveness of using wearable sensors and ML algorithms to accurately classify shoulder rehabilitation exercises. These findings suggest promising prospects for implementing the proposed wearable system in remote home‐based monitoring scenarios. The ease of setup and modularity of the system reduce user burden enabling patient‐driven sensor positioning.

**Level of Evidence:**

Level III.

**Abbreviations**
10 × 5 NCV: 10 outer folds and three inner folds of the nested cross‐validation
5 × 3 NCV: five outer folds and three inner folds of the nested cross‐validation
5 × 5 NCV: fivefolds for both the outer and inner cycles of the nested cross‐validation
LR: Logistic Regression
ML: machine learning
M‐IMU: Magneto‐inertial Measurement Unit
NCV: nested cross‐validation
RC: rotator cuff
RF: Random Forest
ROM: range of motion

## INTRODUCTION

Shoulder disorders represent the most frequently reported musculoskeletal diseases [22, 34], leading to pain, limited range of motion (ROM), decreased functional abilities and quality of life [12, 13, 21]. Exercise therapy is crucial for restoring shoulder functionality and mitigating the likelihood of retear [14, 24, 33]. Traditional shoulder rehabilitation methods involve one‐to‐one sessions with a physiotherapist. Despite all the advantages they provide, these methods have limitations in terms of accessibility, cost and time commitment, leading to the exploration of tele‐rehabilitation as a viable alternative [11, 15, 34]. This approach offers numerous advantages, overcoming geographical barriers, enhancing accessibility to specialized care and promoting patient autonomy [6]. The employment of this technology allows the evaluation of patient adherence to the prescribed exercise programme and the tracking of patient functioning and recovery throughout rehabilitation sessions [7, 17]. This is important because nonadherence to the prescribed rehabilitation programme can result in a prolonged treatment duration and an increased risk of relapse. Several sensors have been introduced to provide objective and quantitative data on body postures, movements and physiological state during physiotherapy sessions [31], providing clinicians with valuable information to assess the effectiveness of the patient's rehabilitation and adapt the on‐going programme as necessary. This facilitates the tailoring of rehabilitation programmes according to individual patient needs, thereby enhancing the overall efficacy of rehabilitative treatments. Magneto‐inertial measurement units (M‐IMUs) have emerged as promising tools for capturing kinematic data, enabling objective analysis of exercise performance [4, 5, 9]. The advent of artificial intelligence (AI) and machine learning (ML) is revolutionizing the healthcare sector. AI algorithms can provide insights into an individual's health status, automatically evaluating exercise execution by estimating pose and joint angles. This enables to give optimal feedback to the patients and tailor rehabilitation treatment plans accordingly [19, 23].

The objective of this study is to train and test ML models to automatically classify shoulder physiotherapy exercises.

## METHODOLOGY

### Data acquisition

This study included both healthy subjects and patients. Patients were selected based on specific criteria, including a diagnosis of rotator‐cuff (RC) tear and completion of at least 6 months of rehabilitation treatment. This study was reviewed and approved by the Ethical Committee of University Campus Bio‐Medico of Rome (protocol code: 15.21 OSS ComEt UCBM). The experimental protocol consisted of six shoulder rehabilitation exercises selected from the guidelines developed by the American Society of Shoulder and Elbow Therapists: [35] Task (1) upright active flexion/extension without any weight; Task (2) upright active flexion/extension using a 2 kg weight; Task (3) external rotation with the shoulder at 90° of abduction using a 2 kg weight; Task (4) towel slide; Task (5) external/internal rotation self‐assisted with a stick and Task (6) abduction/adduction.

Data collection was performed by the same investigators for each participant. The exercises were performed using the volunteers' dominant arm and the patients' pathological limb. The experimenter provided verbal instructions and a practical demonstration of the required movement. Participants underwent a practice trial of each exercise before starting the recording. Each subject performed six consecutive repetitions of each task at a comfortable and self‐selected speed under supervision.

**Figure 1:**
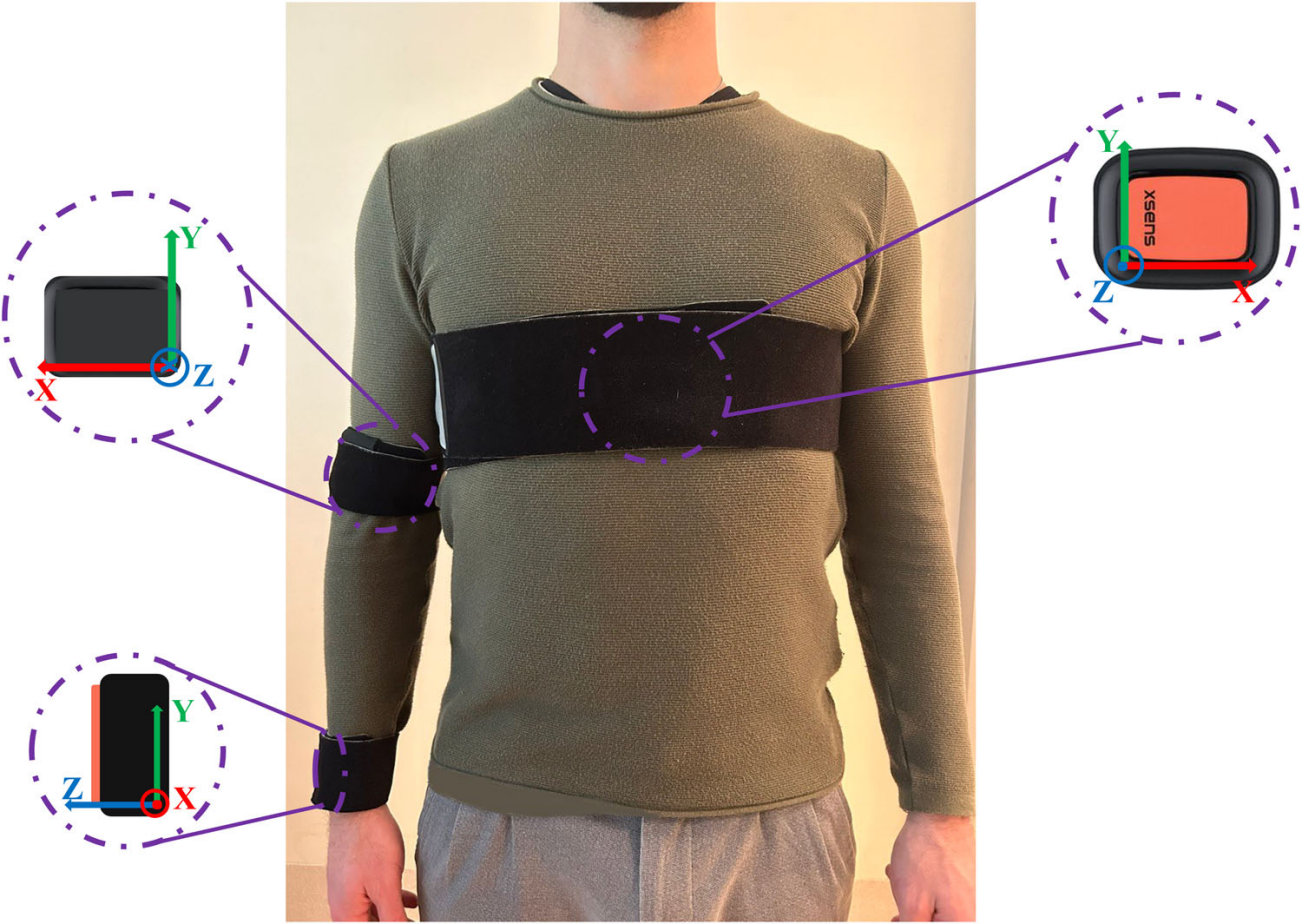
Xsens DOT placement. Each sensor was initially placed horizontally inside the corresponding strap's pocket, with the *y* axis pointing upwards. Subsequently, the three straps were securely wrapped around the segments of interest of thorax, upper arm and forearm. The circles show the coordinate systems of the three sensors: red, green and blue arrows represent *x* axis, *y* axis and *z* axis, respectively. The dot indicates an outgoing arrow, while the cross indicates an incoming arrow.

A wearable system equipped with three M‐IMUs (Xsens DOT, Xsens Technologies) was used in this study. With a dimension of 36.3 × 30.4 × 10.8 mm (length × width × height) and a weight of 10.8 g, these sensors are unobtrusive, allowing unhindered subject movement. Figure 1 illustrates the positions of the sensors on the thorax, upper arm and forearm. The sensors were calibrated before data collection to ensure data accuracy.

### Data preprocessing

**Figure 2:**
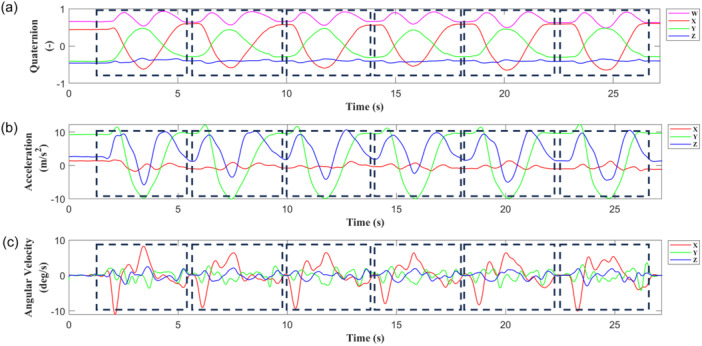
Signal segmentation of (a) quaternion data, (b) accelerometer data, (c) angular velocity data, acquired by forearm Magneto‐inertial Measurement Unit during the six repetitions of Task 1.

During the experiments, three signals were collected for each sensor at a sampling frequency of 60 Hz: delta angle, delta velocity and three‐dimensional orientation (expressed as quaternions). A low‐pass fifth‐order Butterworth filter with a cut‐off frequency of 2 Hz was applied to the data to remove any erroneous or noisy data points. Angular velocity and acceleration were derived from the filtered delta angle and delta velocity data, respectively. A manual segmentation was conducted to isolate individual repetition performed by each participant (Figure 2). Each isolated repetition of each task was considered as a sample, resulting in 1296 overall.

### Features engineering

Subsequently, a feature extraction procedure was performed to capture relevant information that plays a critical role in the classification process [30]. Specifically, the following features were extracted for each sample from each component of the triaxial acceleration data, the triaxial angular velocity data and the quaternion data: variance, mean, standard deviation, median, maximum value, minimum value, range, root mean square, interquartile range (between 25th and 75th percentiles), correlation coefficient, kurtosis and skewness. All features were scaled to zero mean and unit variance to ensure consistency in scale and units [10, 30]. Feature selection aims to reduce the number of features by identifying those that provide highly informative insights into the classification process [20]. Specifically, the principal component analysis was employed as dimensionality‐reduction technique [2, 10, 32]. During data analysis of this study, the components that explained 98% of the variance were selected as features.

### ML models

Six traditional ML models were trained to classify shoulder rehabilitation exercises: *k*‐Nearest Neighbours, Support Vector Machine, Random Forest (RF), Logistic Regression (LR) and Adaptive Boosting with the LR as base estimator [26, 29]. The grid search procedure was used for hyperparameter optimization during the modelling stage of the ML classifiers implementing the nested cross‐validation (NCV) method (see Supporting Information for details) [28, 30, 36]. The entire data set (1296 samples) was divided into two parts, the training set and the testing set [18, 25]. Classifier's performances were evaluated using a confusion matrix, along with various metrics derived from it: accuracy, specificity, sensitivity or recall, precision and F1‐score [1, 25, 30].

## RESULTS

### Cohort demographics

The healthy group consisted of 19 subjects with no shoulder musculoskeletal disorders and no history of upper extremity injuries. During the study period, out of a total of 19 patients, 17 met the eligibility criteria and were, therefore, enrolled in the study. Table 1 shows the characteristics of the participants categorized in the two groups. The age range was 22–28 years for the healthy group and 45–76 years for the patient group. All the included patients were diagnosed with complete RC tears.

**Table 1:** Characteristics of the study sample.

Characteristics	Healthy subjects	Patients
Sex (male/female, no.)	5/14	9/8
Age (years, mean ± standard deviation)	25.18 ± 1.67	61.76 ± 8.87
Height (cm, mean ± standard deviation)	167.89 ± 8.53	166.76 ± 7.73
Weight (kg, mean ± standard deviation)	61.58 ± 11.95	76.88 ± 14.12
BMI (kg/m^2^, mean ± standard deviation)	21.67 ± 2.57	27.63 ± 4.85
Dominant arm (right/left, no.)	19/0	16/1
Pathological side (right/left, no.)	N/A	17/0
Treatment (surgical/conservative, no.)	N.A.	13/4

### Classification performance

The testing set was composed of data from three patients (108 samples) randomly selected from the entire data set. The data of the healthy participants and the remaining 14 patients were included in the training data set. A comparison of the performances was conducted by training the models with different folds' combinations in the NCV: fivefolds for both the outer and inner cycles (5 × 5 NCV), five outer folds and three inner folds (5 × 3 NCV) and 10 outer folds and five inner folds (10 × 5 NCV). Results point out high recognition performances in all cases (see Supporting Information). Implementing the 5 × 5 NCV, the accuracy, F1 score, sensitivity, specificity and precision values ranged from 78.70% to 87.04%, 79.92% to 87.35%, 78.70% to 87.04%, 95.74% to 97.41% and 83.13% to 88.06%, respectively. The decrease of the number of inner folds to three (5 × 3 NCV) did not significantly affect the performance of the classifiers. In this case, the accuracy, F1 score, sensitivity, specificity and precision values were in the range of 74.07%–87.04%, 74.03%–87.30%, 74.07%–87.04%, 94.81%–97.41% and 74.68%–87.96%, respectively. Increasing the number of outer folds by implementing the 10 × 5 NCV led to an improvement in performances' classifiers. For this folds' combination, the accuracy, F1 score, sensitivity, specificity and precision values were in the range of 74.07%–89.81%, 75.33%–89.89%, 74.07%–89.81%, 94.81%–97.96% and 79.38%–90.44%, respectively. Among all cases and across all six models, the RF outperformed the other classifiers across all the metrics, achieving 89.81% of overall accuracy and sensitivity, 89.89% of F1‐score, 97.96% of specificity and 90.44% of precision. The global confusion matrices provide a more detailed insight into the classifiers' performance, allowing the computation of individual accuracy values for each class (see Supporting Information). Averaged accuracies ranged from 92.90% to 95.68% with the 5 × 5 NCV, from 91.36% to 95.68% with the 5 × 3 NCV and from 91.36% to 96.60% with the 10 × 5 NCV.

## DISCUSSION

This study reports the performance of supervised ML models in classifying shoulder rehabilitation exercises using inertial data acquired with three M‐IMUs sensors. The highest performance in classifying the six shoulder exercises was achieved by the RF model, who achieves 89.81% of overall accuracy, 89.89% of F1‐score and 96.60% of averaged accuracy. It is important to acknowledge that the outcomes presented herein pertain to the classification of movements executed by patients with RC tears. Naturally, these patients will exhibit greater variability in the pace and trajectory of their movements, thereby posing increased challenges for the trained classifiers involved in the classification process. Nonetheless, the aforementioned results provide compelling evidence for the effectiveness of the models in effectively learning the patterns and distinguishing features from the collected signal data. The findings of this study align with those of other research studies that utilize IMU sensors for classifying rehabilitation exercises. Accuracies of 96.85% and 96.4% were achieved in two other studies that employed IMU sensors to classify exercises performed by patients [4, 27]. Differently, other studies on the same research topic have solely collected data from healthy subjects [3, 8, 16]. Out of the six exercises, Tasks 4 and 5 exhibited the highest prediction accuracy due to their distinctiveness. Many classifiers occasionally misclassified Task 3, possibly due to its complex execution with the shoulder at 90° of abduction and the elbow flexed at 90°, while holding a weight. Therefore, its execution was more challenging for many patients. The exercises with the lowest prediction accuracy were Tasks 1 and 2, that is, the movements of flexion/extension without and with a weight, respectively. This outcome was foreseeable due to their similar execution in terms of the starting and ending points of the activity (ROM), differing solely in the use of a dumbbell. All participants were instructed to perform these exercises up to their maximal achievable extension. Different from healthy participants, who could complete these exercises up to the maximum extent, some patients exhibited limited ROM due to their pathology. This reduction in ROM was particularly evident during the execution of Task 2. This slight variation in task execution was reflected in the signals and consequently in the extracted features, causing confusion among the classifiers. An IMU sensor gathers data on its position and acceleration. Consequently, using only IMU sensors can be challenging when the position of the sensor on the human body and the movement direction are very similar, despite representing distinct movements (Tasks 1 and 2). Further research will focus on integrating additional sensors, such as electromyography (EMG) sensors. EMG integration provides additional features, such as muscle activation patterns and muscle fatigue, which could enhance classification performances.

Rehabilitation treatment of shoulder disorders is crucial for facilitating a return to daily life and preventing the chronicity of the condition. While home‐based rehabilitation programmes are increasingly used, patients often struggle to perform exercises independently. This study poses the basis for the application of a wearable device for remote monitoring of rehabilitation sessions in the home environment. The application of ML models to classify shoulder rehabilitation exercises shows promise for improving the monitoring of exercise execution and patient adherence to the prescribed programmes. This approach provides benefits to clinicians, enabling them to monitor patient's exercise records and tailor the programmes based on their performance. Conversely, patients can be more motivated by receiving professional feedback even when they are at home. This can optimize the treatment and recovery process, improving outcomes in shoulder pain rehabilitation programmes.

Some limitations are evident in this study. First, only six common rehabilitation exercises were selected. Additional movement exercises will be explored in future studies to further validate the algorithm's appropriateness. Second, since the experiments were conducted under controlled laboratory conditions, future work will investigate the model's applicability in unsupervised settings. The third limitation of the study concerns the absence of an objective evaluation of exercise performance. By remotely assessing patients' performance, researchers and therapists can gain better insights into the effectiveness of rehabilitation programmes, aiding in personalized treatment for each patient. This will enhance the quality of care for patients with shoulder musculoskeletal conditions, improving their rehabilitation outcomes.

## CONCLUSIONS

The integration of ML into technological systems is revolutionizing health care by enhancing patient care and increasing efficiency. Specifically, the classification of shoulder rehabilitation exercises holds significant potential for improving the monitoring of exercises and patient adherence to the prescribed programmes. The findings of this study hold promise for the potential application of the proposed wearable system for home‐based remote monitoring. The proposed system, equipped with wireless sensors and characterized by an easy and fast setup, reduces user burden, enabling patient‐driven sensor positioning.

## AUTHOR CONTRIBUTIONS

All authors contributed to the conception and design of the study. Material preparation and data collection were performed by Martina Sassi and Matilde Mancuso. Martina Sassi analysed data and wrote the manuscript. All authors read, reviewed and approved the final version of the manuscript.

## CONFLICT OF INTEREST STATEMENT

The authors declare no conflict of interest.

## ETHICS STATEMENT

The study was conducted in accordance with the Declaration of Helsinki. Ethical approval has been gained from the local Ethical Committee of University Campus Bio‐Medico of Rome (protocol code 15.21 OSS ComEt UCBM). Patients signed informed consent regarding publishing their data and photographs. Informed consent was obtained from all participants included in the study.

## Supporting information

Supporting Information.

Supporting Information.

Supporting Information.

Supporting Information.

Supporting Information.

Supporting Information.

Supporting Information.

Supporting Information.

Supporting Information.

Supporting Information.

## Data Availability

The data presented in this study are available on request from the corresponding author.
