# Supplementary material for: Machine‐learning models for shoulder rehabilitation exercises classification using a wearable system
Source: Knee Surg Sports Traumatol Arthrosc. 2024 Aug 18;33(4):1452–8. doi: 10.1002/ksa.12431 (PMC11948177; doi:10.1002/ksa.12431)
Supplement: Supplementary file 1 — Supporting Information. [file KSA-33-1452-s009.docx]

Definition of grid search space for tuning the hyper-parameters of the implemented classifiers.

| **Supervised Classifiers** | **Hyper-parameters** | **Grid Search space** | **Description** |
| --- | --- | --- | --- |
| **Support Vector Machine** | C | From 0.001 to 10, with 50 samples evenly distributed logarithmically along this interval. | Regularization parameter which has a significant effect on the generalization performance of the classifier. |
|  | Kernel | Linear, polynomial, radial basis function, sigmoid | Type of kernel function used in the algorithm in order to transform the input data into a higher-dimensional space. |
| **k-Nearest Neighbours** | Number of neighbours | From 1 to 19 | Number of neighbours |
|  | Algorithm | Ball-tree; KD-tree; Brute. | Algorithm used to compute the nearest neighbours. |
| **Decision Tree** | Criterion | Gini index, Entropy, Log-loss | Function used to evaluate the quality of a split during the tree building process. |
| **Random Forest** | Number of trees | From 30 to 500, with step size 10 | Number of decision trees in the forest. |
|  | Criterion | Gini index, Entropy, Log-loss | Function used to evaluate the quality of a split when building individual decision trees within the forest. |
| **Logistic Regression** | C | From 0.001 to 100, with 100 samples evenly distributed logarithmically along this interval. | Regularization parameter that controls the inverse of the regularization strength. |
|  | Solver | Lbfg (Limited-memory Broyden–Fletcher–Goldfarb–Shanno); newton-cg; Stochastic Average Gradient (SAG); SAGA. | Algorithm use in the optimization problem. |
| **Adaptive Boosting** | Number of base estimators | 10, 50, 100, 150, 200 | The maximum number of estimators that can be used to build the ensemble learning model. |
|  | Learning rate | 0.001, 0.01, 0.1, 1 | Regularization parameter that scales the contribution of each estimator. |
