# Supplementary material for: Machine‐learning models for shoulder rehabilitation exercises classification using a wearable system
Source: Knee Surg Sports Traumatol Arthrosc. 2024 Aug 18;33(4):1452–8. doi: 10.1002/ksa.12431 (PMC11948177; doi:10.1002/ksa.12431)
Supplement: Supplementary file 3 — Supporting Information. [file KSA-33-1452-s003.docx]

Metrics of performance of all classifiers implementing the nested cross validation method with 5 outer folds and 3 inner folds.

| **Classifiers** | **Accuracy** | **F1 score** | **Sensitivity** | **Specificity** | **Precision** |
| --- | --- | --- | --- | --- | --- |
| k-NN | 0.8241 | 0.8321 | 0.8241 | 0.9648 | 0.8462 |
| SVM | 0.7778 | 0.7915 | 0.7778 | 0.9556 | 0.8285 |
| DT | 0.7407 | 0.7403 | 0.7407 | 0.9481 | 0.7468 |
| RF | 0.8241 | 0.8295 | 0.8241 | 0.9648 | 0.8412 |
| LR | 0.8704 | 0.8730 | 0.8704 | 0.9741 | 0.8796 |
| AB | 0.8241 | 0.8281 | 0.8241 | 0.9648 | 0.8389 |

k-NN: k-Nearest Neighbour; SVM: Support Vector Machine; DT: Decision Tree; RF: Random Forest; LR: Logistic Regression; AB: Adaptive Boosting.
