# Supplementary material for: Machine‐learning models for shoulder rehabilitation exercises classification using a wearable system
Source: Knee Surg Sports Traumatol Arthrosc. 2024 Aug 18;33(4):1452–8. doi: 10.1002/ksa.12431 (PMC11948177; doi:10.1002/ksa.12431)
Supplement: Supplementary file 5 — Supporting Information. [file KSA-33-1452-s001.docx]

Recognition accuracy for all 6 shoulder exercises implementing the nested cross validation method with 5 outer and 5 inner folds.

| **Classifiers** | **FE** | **FEd** | **ERs** | **SL** | **EIR** | **AA** | **Averaged accuracy** |
| --- | --- | --- | --- | --- | --- | --- | --- |
| k-NN | 0.8426 | 0.8519 | 1 | 0.9907 | 0.9907 | 0.9722 | 0.9414 |
| SVM | 0.8426 | 0.8148 | 0.9444 | 1 | 1 | 0.9722 | 0.9290 |
| DT | 0.8889 | 0.8519 | 0.9630 | 1 | 0.9722 | 0.9352 | 0.9352 |
| RF | 0.8889 | 0.8889 | 0.9815 | 1 | 0.9907 | 0.9907 | 0.9568 |
| LR | 0.8889 | 0.8981 | 0.9815 | 1 | 1 | 0.9722 | 0.9568 |
| AB | 0.8426 | 0.8333 | 0.9815 | 1 | 1 | 0.9907 | 0.9414 |

k-NN: k-Nearest Neighbour; SVM: Support Vector Machine; DT: Decision Tree; RF: Random Forest; LR: Logistic Regression; AB: Adaptive Boosting; FE: Upright flexion/extension without a weight; FEd: Upright flexion/extension with a weight (2 kg); ERs: External rotation with the shoulder at 90° of abduction, holding a weight (2 kg); SL: Towel slide; EIR; External/internal rotation self-assisted with a stick; AA: Abduction/adduction.
