# Supplementary material for: Machine‐learning models for shoulder rehabilitation exercises classification using a wearable system
Source: Knee Surg Sports Traumatol Arthrosc. 2024 Aug 18;33(4):1452–8. doi: 10.1002/ksa.12431 (PMC11948177; doi:10.1002/ksa.12431)

Confusion matrices for activity recognition using the nested cross validation method with 5 outer and 3 inner folds. Shoulder exercises are as follow, 1: flexion/extension without a weight; 2: flexion/extension with a weight; 3: External rotation with the shoulder at 90° of adduction, holding a weigh; 4: Towel slide; 5: External/internal rotation self-assisted with a stick; 6: Abduction/adduction. (a), (b), (c), (d), (e), and (f) represent confusion matrices for the k-NN classifier, DT classifier, LR classifier, SVM classifier, RF classifier, and AB classifier, respectively


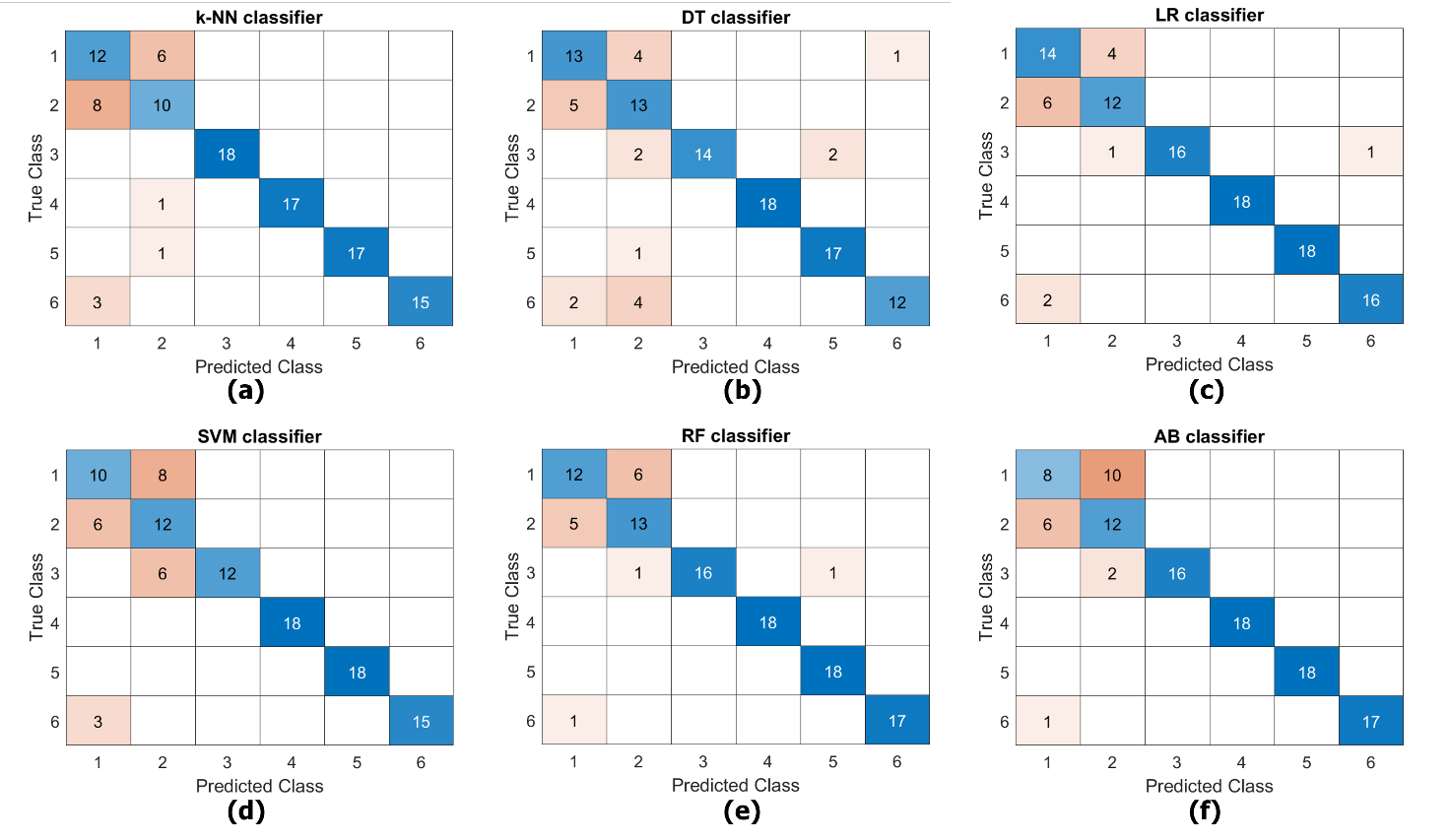

Supplement: Supplementary file 8 — Supporting Information. [file KSA-33-1452-s010.docx]
